# Maternal health literacy as a potential determinant of infant and early childhood health: a systematic review

**DOI:** 10.3389/fpubh.2026.1743880

**Published:** 2026-04-15

**Authors:** Zanmei Li, Jiao Li, Weiwei Yao, Liangkun Ma

**Affiliations:** 1 Institute of Medical Information and Library, Chinese Academy of Medical Sciences and Peking Union Medical College, Beijing, China; 2 Beijing MYYA Health Management Co., Ltd., Beijing, China; 3 Department of Obstetrics and Gynecology, Peking Union Medical College Hospital, Beijing, China

**Keywords:** behavioral pathways, early childhood health, health equity, maternal health literacy, public health determinants, systematic review

## Abstract

**Background:**

Maternal health literacy (MHL) represents a potentially modifiable public health determinant concentrated among socioeconomically disadvantaged populations. While 15–45% of pregnant women demonstrate inadequate health literacy, its impact across the full spectrum of infant and early childhood outcomes remains inadequately characterized, limiting understanding of its broader public health significance.

**Objective:**

To systematically synthesize evidence on associations between MHL and infant/child health outcomes from birth through age three, and to examine whether MHL functions as a systemic determinant across outcome domains.

**Methods:**

Following PRISMA guidelines, we searched MEDLINE, Embase, and Web of Science from inception through March 2025. Studies examining associations between validated MHL measures and any child health outcomes up to age three were included. Methodological quality was assessed using JBI checklists, and the certainty of evidence using the GRADE framework. Narrative synthesis was conducted.

**Results:**

Eight studies (*n* = 13,407 participants) from seven countries met inclusion criteria. Higher MHL was consistently associated with favorable birth weight. A pattern consistent with a behavioral pathway hypothesis emerged: outcomes requiring maternal behavioral competencies (e.g., symptom recognition, care-seeking) showed stronger and more consistent associations (e.g., reduced neonatal jaundice readmission, diaper dermatitis) than medically-determined conditions. Limited evidence also suggested MHL effects beyond the perinatal period, with inadequate MHL associated with increased risks of nutritional deficiencies and developmental delays (over four-fold increased risk in vulnerable populations). JBI assessment indicated generally adequate quality, while GRADE rated the certainty of evidence as Low to Very Low due to cross-sectional designs and imprecision.

**Conclusion:**

MHL shows promising systematic associations with infant outcomes across multiple domains spanning the early life course, operating primarily through behavioral pathways requiring maternal agency. This pattern supports conceptualizing MHL as a potential foundational public health determinant and equity-relevant intervention target. However, low certainty of evidence underscores the need for standardized measurement, longitudinal studies, and intervention trials to establish causality.

## 1 Introduction

Health literacy (HL) has emerged as a fundamental health determinant, defined as the cognitive and social competencies enabling individuals to access, understand, appraise, and apply health information to promote health (1, 2). Contemporary understanding positions health literacy as a systemic determinant operating at the intersection of individual capabilities, health system complexity, and social-environmental contexts (3–5). Evidence demonstrates health literacy may be a stronger health predictor than traditional socioeconomic indicators including income, education, and employment status (6, 7). Individuals with limited health literacy consistently demonstrate poorer health service utilization, reduced preventive care engagement, lower medication adherence, increased hospitalization frequency, and elevated healthcare costs (8–11).

Pregnancy and early motherhood represent a uniquely critical period for public health intervention, characterized by increased health information needs, frequent healthcare encounters, heightened motivation for behavior change, and consequential decision-making affecting both maternal and infant wellbeing (12). Maternal health literacy (MHL)—the constellation of skills enabling pregnant and postpartum women to access, understand, evaluate, and effectively use pregnancy-specific and child health information—emerges as a potentially powerful determinant of maternal agency and caregiving quality (13–17). Population-based studies indicate 30–50% of adults possess inadequate HL, with disparities pronounced among pregnant women from disadvantaged backgrounds (17–21). From a health equity perspective, this concentration positions MHL as a promising intervention target: because limited health literacy is prevalent among disadvantaged groups yet potentially modifiable, successful enhancement strategies could simultaneously improve outcomes and reduce disparities (22).

Emerging evidence demonstrates associations between inadequate MHL and adverse maternal outcomes and behaviors, including delayed prenatal care, poor pregnancy self-management, increased cesarean rates, suboptimal breastfeeding, and delayed vaccination (23–27). However, a critical evidence gap remains: while existing research has documented associations between MHL and maternal behaviors (intermediate outcomes), the direct relationship between MHL and actual infant health status outcomes (ultimate endpoints) remains inadequately characterized. Moreover, existing research tends to examine MHL’s influence on isolated outcomes rather than assessing its potential function as a cross-cutting determinant influencing multiple health domains through shared mechanisms (16, 28).

This systematic review addresses these critical evidence gaps by providing the first comprehensive synthesis examining associations between MHL and the full spectrum of infant and early childhood health outcomes across the first 3 years of life. By deliberately employing broad, inclusive outcome criteria, this review aims to empirically examine whether MHL functions as such a systemic determinant. Our objectives are to: (1) Map the comprehensive scope of infant/child health outcomes examined in relation to MHL; (2) Synthesize evidence on associations between MHL and outcomes across diverse domains; (3) Explore potential mechanistic patterns through which MHL may influence different outcome categories; (4) Evaluate methodological quality and limitations; (5) Inform public health policy by clarifying whether MHL functions as a systemic determinant justifying broad intervention investment.

## 2 Methods

### 2.1 Search strategy

This systematic review followed the Preferred Reporting Items for Systematic Reviews and Meta-Analyses (PRISMA) guidelines (29). The protocol for this systematic review was not prospectively registered in PROSPERO, however, methodological rigor was ensured through strict adherence to PRISMA guidelines and predefined eligibility criteria and analytic procedures. Comprehensive literature searches were performed across MEDLINE (via PubMed), Embase, and Web of Science databases from inception through March 5th, 2025. The search strategy was developed through iterative consultation with an experienced medical librarian. Importantly, outcome search terms were deliberately kept broad and inclusive to capture the full spectrum of infant health indicators potentially influenced by MHL, avoiding premature restriction that might bias synthesis toward specific outcome categories.

The strategy combined Medical Subject Headings (MeSH) terms and free-text keywords related to maternal health literacy and infant outcomes using Boolean operators. Primary search terms included: “health literac*,” “maternal,” “pregnan*,” “perinatal,” “postpartum,” combined with broad outcome terms including “infant,” “newborn,” “child*,” “neonat*,” “birth,” “development*,” “growth,” “health outcome*.” Searches were limited to English-language studies. Reference lists of included studies and relevant reviews were manually searched. The detailed search strategy is presented in Appendix 1.

### 2.2 Eligibility criteria

Studies were included based on a modified PICOS framework (30) designed to capture MHL’s comprehensive influence:

*Population*: pregnant women, postpartum women, or mothers of infants and young children under 3 years. No restrictions on maternal age, parity, gestational age, or complications to maximize generalizability.
*Intervention/Exposure*: Validated or structured measurement instruments with established psychometric properties. Both pregnancy-specific and general population health literacy instruments applied during pregnancy or the postpartum period were acceptable. Studies employing unstandardized questionnaires, single-item measures, or proxy indicators (such as educational attainment alone) were excluded.
*Comparator*: Health literacy examined as continuous or categorical variable, or comparing different levels.
*Outcomes*: At least one infant or child health outcome up to 3 years. We employed deliberately inclusive outcome criteria to map MHL’s full scope of influence across three interconnected domains: (1) Biological/Birth Indicators: birth weight, gestational age, growth parameters, Apgar scores, mode of delivery; (2) Acute Neonatal Complications: NICU admission, respiratory support, feeding difficulties, jaundice, infections, dermatological conditions—encompassing both medically-determined complications and conditions influenced by maternal recognition/caregiving; (3) Longer-term Growth and Development: anthropometric measures, nutritional status (stunting, wasting, underweight), developmental milestones, behavioral development.
This broad, multi-dimensional outcome framework was essential: rather than restricting to pre-specified categories based on theoretical assumptions, we sought to empirically examine whether MHL demonstrates systematic associations across diverse domains or whether effects cluster in specific types.
*Study design*: Observational studies (cross-sectional, prospective cohort, retrospective cohort, case–control) and experimental studies (randomized controlled trials, quasi-experimental studies). Case reports, case series, narrative reviews, systematic reviews, and conference abstracts were excluded.
*Analysis*: Quantitative results examining statistical associations between MHL and outcomes. Appropriate methods include correlation analysis, regression modeling, chi-square tests, or other inferential approaches. Purely descriptive studies without statistical testing of associations were excluded.

#### Selection of studies

All identified records were imported to reference management software for duplicate removal. Title/abstract screening and full-text screening were conducted independently by two reviewers (ZL & WY). Disagreements were resolved through discussion, with third reviewer consultation (LM) when necessary.

### 2.3 Data extraction

Data were extracted independently by reviewers (ZL and WY) using a standardized form pilot-tested on two studies (31, 32). Extracted information included: study characteristics (author, year, country, sample size, design), participant details (maternity stage, demographics), health literacy assessment (instrument, timing, categorization), infant/child outcomes (specific outcomes, timing, measurement method), statistical methods, effect estimates, and confounders adjusted. Discrepancies were double-checked in original articles. To enable meaningful comparison, MHL levels were standardized into three categories: ‘Limited’ (inadequate, insufficient, low, problematic), ‘Marginal’ (medium, fair), and ‘Adequate’ (sufficient, high, excellent).

### 2.4 Quality assessment

Methodological quality was independently assessed by two reviewers using appropriate Joanna Briggs Institute (JBI) Critical Appraisal Checklists: the JBI Checklist for Analytical Cross-Sectional Studies (8 items) for cross-sectional studies, and the JBI Checklist for Randomized Controlled Trials (13 items) for RCTs (33). Both checklists were scored with ‘Yes’, ‘No’, ‘Unclear’, or ‘Not Applicable’. A third reviewer (ML) resolved disagreements. Furthermore, the certainty of evidence for each infant health outcome was evaluated using the Grading of Recommendations Assessment, Development and Evaluation (GRADE) framework (34). The certainty was classified into four levels—high, moderate, low, or very low—based on five domains: risk of bias, inconsistency, indirectness, imprecision, and publication bias. Detailed JBI item-by-item results are provided in Appendix 2, and the complete GRADE evidence profile is presented in Appendix 3.

### 2.5 Data synthesis

Due to substantial heterogeneity in study designs, health literacy measurement instruments, outcome definitions, and statistical reporting, meta-analysis was not methodologically appropriate. Narrative synthesis was conducted following established guidelines, organizing findings by three outcome domains:

(1) *Birth outcomes and immediate indicators*: birth weight, gestational age, Apgar scores, preterm birth, low birth weight, macrosomia, small-for-gestational-age—reflecting immediate fetal health and development;
(2) *Neonatal complications and acute health events*: NICU admission, respiratory support, jaundice requiring readmission, diaper dermatitis—reflecting both medical necessities and complications influenced by maternal recognition and care-seeking;
(3) *Postnatal growth and development*: anthropometric indices, nutritional status (stunting, underweight, wasting), developmental evaluations—reflecting cumulative effects of ongoing caregiving.

This domain-based synthesis structure enables examination of whether association patterns differ systematically by outcome type and facilitates identification of potential mechanistic pathways through which MHL influences different categories of infant outcomes. For outcomes where multiple studies reported consistent findings, results are presented in tabular form; outcomes examined in single studies or yielding null findings are described narratively.

## 3 Results

### 3.1 Study selection

**Figure 1:**
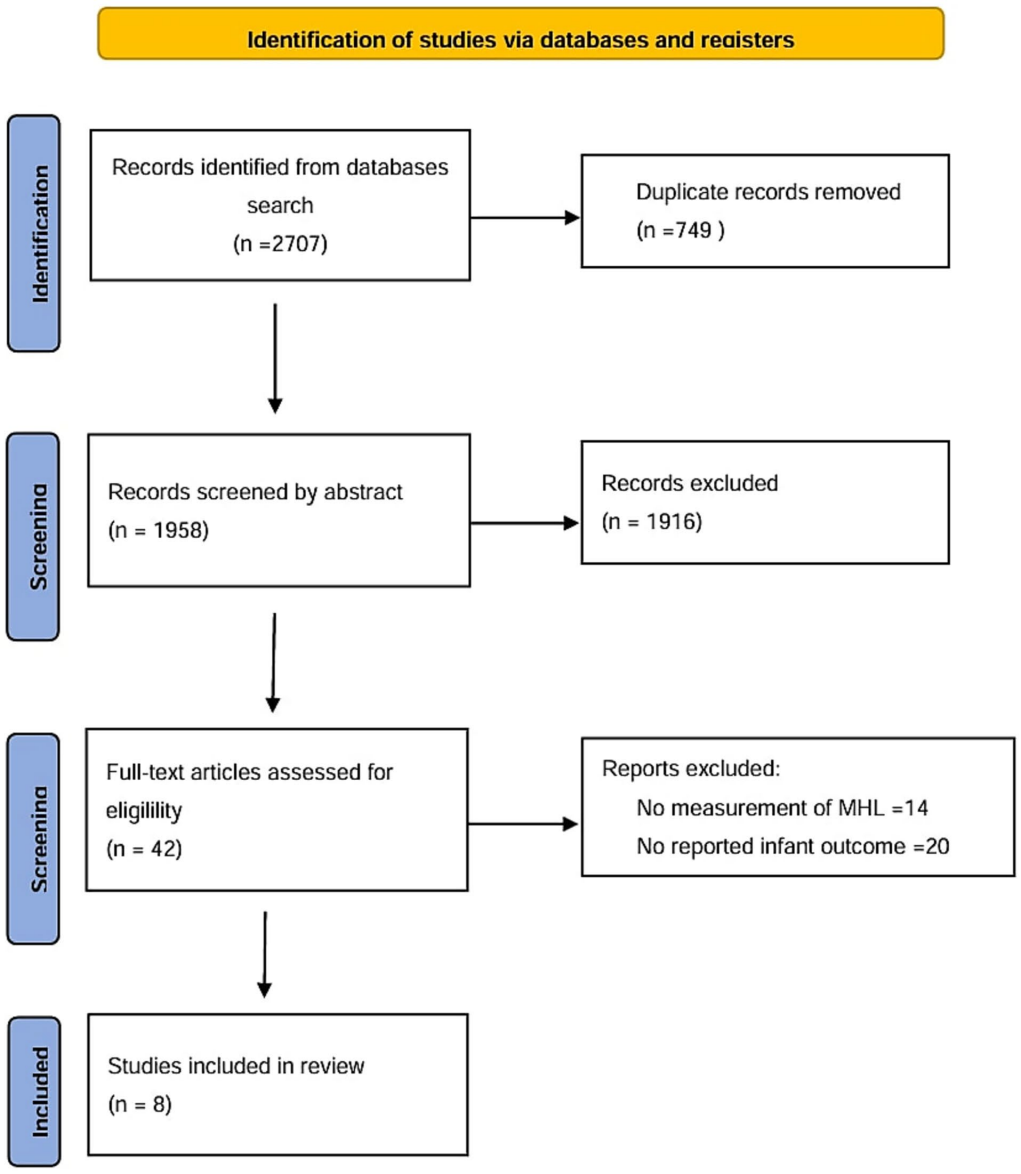
PRISMA study selection flowchart.

The systematic search yielded 2,707 records. Following duplicate removal, 1,958 unique records underwent title and abstract screening, resulting in 42 studies for full-text review. Rigorous eligibility assessment excluded 34 studies: primary due to non-validated health literacy instrument (n = 14) and failure to report relevant infant health outcomes (*n* = 20). Manual reference searching yielded no additional studies. Ultimately, eight studies (*n* = 13,407 participants) from seven countries met all inclusion criteria and were included (24, 31, 32, 35–39). The low overall yield is itself informative about the current state of the field: the two dominant exclusion patterns—prevalent use of non-validated MHL proxies (*n* = 14) and the field-wide focus on maternal behavioral intermediates rather than direct infant health endpoints (*n* = 20)—reveal precisely the methodological gaps that motivated this review and that future research must address. The complete process is detailed in the PRISMA flowchart (Figure 1).

### 3.2 Study characteristics

The eight included studies demonstrated notable diversity in terms of temporal distribution, geographic representation, and methodological approaches (Table 1). The included studies varied substantially in study design, measurement instruments, and outcome definitions, contributing to methodological heterogeneity across the evidence base.

**Table 1:** Characteristics of included studies.

Study	Country/region	Sample size	Study design	MHL instrument used	MHL assessment timing	Infant health outcomes assessed
Rahimli et al. (36)	Turkey	188	cross-section study	HLS-EU-Q16	Early second trimester of pregnancy	APGAR scores
Yee et al. (31)	USA	9,341	cross- section study	REALM-SF	16 and 21 weeks’gestation	Small for gestational age, low birth weight, very low birth weight, macrosomia, preterm birth, admission to the neonatal intensive care unit, 5-min Apgar score, and receipt of neonatal respiratory support
Gaupšienė et al. (24)	Lithuania	500	cross- section study	HLS-EU-Q47	Days 2 and 3 after birth	Birth weight, birth height, Apgar score
Izadirad et al. (38)	Iran	860	cross- section study	HELIA, Health literacy of iranian adults	Between 14 and 25 weeks	Birth weight
Hernandez-Mekonnen et al. (35)	USA	87	cross- section study	PHLAT-8-Spanish version	Children 12–60 months of age	Developmental delay (ASQ-3 screener)
Johri et al. (32)	India	1773	cross- section study	Self-developed instrument	Children aged 12–23 months	Height-for-age (HAZ), weight-for-age (WAZ), and weight-for-height (WHZ)
Eksamut et al. (39)	Thailand	400	cross- section study	Thai health literacy scale	Post-discharge (infants were 4–5 days old)	Neonatal Jaundice admission
Cheng et al. (37)	China	Intervention *n* = 128 Control *n* = 130	randomized controlled trials	Perinatal maternal health literacy scale	At hospital admission for birth	Diaper dermatitis

#### 3.2.1 Temporal trends

Publication dates ranged from 2016 to 2025, with six (75%) published from 2020 onwards, reflecting growing recognition of health literacy’s importance in maternal-child health (40).

#### 3.2.2 Geographic distribution

Research spanned seven countries across diverse economic contexts: three high-income countries (United States n = 2, Lithuania n = 1), four upper-middle-income countries (Turkey, China, Iran, Thailand), and one lower-middle-income country (India). This underscores MHL’s global relevance while highlighting limited research from low-income settings where health literacy challenges may be most pronounced.

#### 3.2.3 Sample sizes and designs

Sample sizes ranged from 87 to 9,341 participants (total n = 13,407). Seven studies employed cross-sectional designs; one was a randomized controlled trial. This predominance of observational research represents a critical limitation for causal inference.

#### 3.2.4 Outcome diversity across the life course

Studies examined outcomes spanning all three pre-defined domains:

- *Birth outcomes* (*n* = 4 studies): birth weight, gestational age, Apgar scores, SGA, preterm birth, macrosomia.
- *Neonatal complications* (*n* = 3): NICU admission, respiratory support, jaundice readmission, diaper dermatitis.
- *Postnatal growth and development* (*n* = 2): stunting, underweight, wasting, developmental delay risk.

This comprehensive coverage—from immediate birth parameters through 23-month developmental milestones—enabled systematic examination of whether MHL demonstrates consistent influence across diverse domains.

### 3.3 Health literacy assessment approaches

Substantial heterogeneity characterized health literacy measurement across the eight included studies, with eight distinct instruments employed and no two studies using identical tools. This diversity reflects evolving theoretical frameworks and the absence of consensus regarding optimal assessment strategies for maternal populations. The diversity of measurement instruments may affect comparability across studies and represents an important methodological limitation of the current evidence base.

#### 3.3.1 Instruments used

- Pregnancy-specific tools: Perinatal Maternal Health Literacy Scale (41), Self-developed instrument (32)
- General population tools adapted for maternal use: HLS-EU-Q47, HLS-EU-Q16, REALM-SF, HELIA (Health Literacy for Iranian Adults) (42), Thai Health Literacy Scale (43), PHLAT-8-Spanish version

#### 3.3.2 Theoretical frameworks

Following Nutbeam’s framework, three studies assessed exclusively functional health literacy, while five employed multidimensional tools measuring functional, interactive, and critical health literacy domains.

#### 3.3.3 Assessment timing

Timing of MHL assessment varied considerably, ranging from the second trimester of pregnancy through several months postpartum. This temporal variation creates uncertainty about optimal measurement windows for different outcomes and may contribute to observed heterogeneity in findings.

#### 3.3.4 Prevalence of limited MHL

The proportion of mothers with limited/inadequate health literacy ranged from 15 to 45% across studies, with higher rates consistently observed among socioeconomically disadvantaged populations. These findings align with population-based estimates indicating 30–50% of adults possess inadequate health literacy globally.

The instruments varied substantially in theoretical grounding, item count, validation context, and suitability for perinatal populations, as summarized in Table 2. Notably, two instruments were developed specifically for the perinatal context: the Perinatal Maternal Health Literacy Scale, which has been formally validated (41), and a self-developed instrument used in one study (32) that, while contextually appropriate, lacks formal psychometric validation. The remaining six tools were general adult health literacy instruments adapted for maternal use. This instrument-level heterogeneity represents a critical structural limitation, as cross-study comparability is constrained when the measured construct itself may differ between tools.

**Table 2:** Comparative characteristics of MHL assessment instruments.

Instrument	Construct type(s)^1^	Validation status/perinatal specificity	Timing in included study	Study
HLS-EU-Q16	Functional, interactive, and critical	Validated/ Non-specific	Early second trimester	Rahimli et al. (36)
REALM-SF	Functional	Validated/Non-specific	16–21 weeks gestation	Yee et al. (31)
HLS-EU-Q47	Functional, interactive, and critical	Validated/Non-specific	Days 2–3 postpartum	Gaupšienė et al. (24)
HELIA	Functional, interactive, and critical	Validated/Non-specific	14–25 weeks gestation	Izadirad et al. (38)
PHLAT-8-Spanish version	Functional	Validated/ Non-specific	12–60 months postpartum	Hernandez-Mekonnen et al. (35)
Self-developed	Functional	Not formally validated/Perinatal- specific	12–23 months postpartum	Johri et al. (32)
Thai health literacy scale	Functional, interactive, and critical	Validated/ Non-specific	Post-discharge (4–5 days)	Eksamut et al. (39)
Perinatal maternal health literacy scale	Functional, interactive, and critical	Validated/Perinatal- specific	hospital admission for birth	Cheng et al. (37)

REALM-SF, Rapid Estimate of Adult Literacy in Medicine–Short Form; HLS-EU, Health Literacy Survey–European Union; HELIA, Health Literacy for Iranian Adults; PHLAT, Perinatal Health Literacy Assessment Tool.

### 3.4 Methodological quality and certainty of evidence

Table 3 presents the methodological quality of included studies and the certainty of evidence for each outcome domain. JBI quality assessment indicated generally adequate quality, with seven cross-sectional studies scoring 5–7 out of 8 points and the RCT scoring 9 out of 13 points. Item-level patterns (Appendix 2) highlight three systematic methodological weaknesses. First, confounder handling was limited: Q6 (confounder management strategies) was unmet in all seven cross-sectional studies, and Q5 (confounder identification) in four of seven, potentially inflating observed associations. Second, the cross-sectional design precludes establishing temporal precedence, leaving the possibility of reverse causation unresolved. Third, Q3 (exposure measurement validity) was unmet in four of seven studies; reliance on non–perinatal-specific instruments may therefore have attenuated the true effects of maternal health literacy (MHL). In contrast, Q7 (outcome measurement validity) was satisfied across all seven studies, representing a relative methodological strength. In the single randomized controlled trial (RCT), incomplete blinding (Q4–Q6) reflects inherent constraints of behavioral interventions and is correspondingly captured in its Low certainty rating under the GRADE framework.

**Table 3:** Quality assessment and certainty of evidence.

Outcome domain	Specific outcome	Included studies	JBI score^1^	GRADE certainty^2^
Birth outcomes	Birth weight	Yee et al. (31) Gaupšienė et al. (24) Izadirad et al. (38)	6/8 6/8 6/8	⊕ ⊕ Low
	Apgar scores	Yee et al. (31) Rahimli et al. (36) Gaupšienė et al. (24)	6/8 6/8 6/8	⊕Very Low
Neonatal complications	Diaper dermatitis	Cheng et al. (37)	9/13	⊕ ⊕ Low
	Jaundice readmission	Eksamut et al. (39)	7/8	⊕Very Low
Growth and development	Nutritional status	Johri et al. (32)	6/8	⊕Very Low
	Development delay	Hernandez-Mekonnen et al. (35)	5/8	⊕Very Low

JBI^1^ score: Number of “Yes” responses over total applicable items (8 items for cross-sectional studies; 13 items for RCTs); GRADE certainty^2^: Evidence downgraded due to observational design, imprecision (small samples), or inconsistency.

The overall certainty of evidence ranged from ‘Low’ to ‘Very Low’ across all outcome domains. Birth weight and diaper dermatitis were rated as ‘Low’ certainty, while Apgar scores, neonatal jaundice, and developmental outcomes were downgraded to ‘Very Low’ certainty primarily due to serious imprecision (small sample sizes) and clinical heterogeneity.

### 3.5 Associations between MHL and infant outcomes

The specific associations identified between MHL and infant outcomes, which should be interpreted alongside the quality and certainty metrics presented in Table 4, are detailed below by domain.

**Table 4:** Summary of findings.

Outcome domain	Specific outcome	Behavioral pathway	Included studies	Key findings and effect size
Birth outcomes	Birth weight	Behavior-dependent	Yee et al. (31) Gaupšienė et al. (24) Izadirad et al. (38)	Inadequate MHL increased low birth weight risk (aRR 1.33, 95% CI 1.07–1.65) and showed an inverse association with adverse outcomes (OR 0.66, 95% CI 0.45–0.95).
	Apgar scores	Medically-determined	Yee et al. (31) Rahimli et al. (36) Gaupšienė et al. (24)	Mixed findings: Significant association in one large US cohort (aRR 2.78, 95% CI: 1.16–6.65); non-significant in others.
Neonatal complications	Diaper dermatitis	Behavior-dependent	Cheng et al. (37)	Significant reduction in intervention group (χ2 = 10.896, *p* = 0.001) compared to standard care.
	Jaundice readmission	Behavior-dependent	Eksamut et al. (39)	MHL was the sole significant predictor of neonatal jaundice readmissions (aOR 0.96, 95% CI 0.94–0.98) for Myanmar mothers
Growth and development	Nutritional status	Behavior-dependent	Johri et al. (32)	Adequate MHL linked to ~50% reduced odds of severe stunting (rural aOR 0.50, 95% CI 0.33–0.74; urban aOR 0.58, 95% CI 0.35–0.94) and underweight (rural aOR 0.57, 95% CI 0.38–0.87; urban aOR 0.48, 95% CI 0.25–0.91).
	Development delay	Behavior-dependent	Hernandez-Mekonnen et al. (35)	Low MHL associated with >4-fold increased risk of developmental delay (aOR 4.4, 95% CI: 1.3–15.4).

Behavior-dependent (outcomes where maternal information processing, symptom recognition, decision-making, or caregiving execution directly influence health status); Medically-determined (outcomes determined primarily by clinical and acute obstetric circumstances independent of maternal behavioral agency). aRR, Adjusted risk ratio; aOR, Adjusted odds ratio; OR, Odds ratio; CI, Confidence interval.

#### 3.5.1 Domain 1: birth outcomes—consistent evidence for birth weight

Birth outcomes demonstrated the most consistent associations with MHL across three studies encompassing diverse populations and settings.

##### 3.5.1.1 Birth weight

Higher MHL was associated with higher newborn birth weight, with mothers having adequate health literacy demonstrating approximately two-fold increased likelihood of delivering normal-weight infants (24). Inadequate MHL was associated with significantly elevated adjusted risk of low birth weight (95% CI 1.07–1.65) (31). Another study confirmed MHL’s effect on birth weight outcomes (OR 0.66, 95% CI 0.45–0.95) (38). These associations persisted after extensive adjustment for sociodemographic confounders including maternal education, income, age, and race/ethnicity—suggesting MHL operates partially independently of traditional socioeconomic pathways.

##### 3.5.1.2 Apgar scores

Findings for Apgar scores were inconsistent. One large US cohort identified inadequate MHL associated with increased odds of 5-min Apgar <4 (31), while two other studies reported no significant relationships (24, 36). This inconsistency likely reflects Apgar scores’ multifactorial nature—influenced by delivery circumstances and acute medical conditions potentially less modifiable through maternal health literacy pathways than cumulative intrauterine growth.

##### 3.5.1.3 Gestational age

No significant association between MHL and preterm birth following adjustment for confounders (31). This null finding contrasts sharply with birth weight associations and suggests that MHL’s influence may be more pronounced for fetal growth parameters than for gestational duration. Preterm birth involves complex etiologies that may be less directly influenced by maternal health behaviors mediated through health literacy mechanisms.

#### 3.5.2 Domain 2: neonatal complications—the behavioral pathway hypothesis

Analysis of neonatal complications revealed the review’s most conceptually significant finding: a distinctive pattern whereby outcomes requiring maternal behavioral competencies showed stronger and more consistent associations with MHL than complications determined primarily by medical factors.

##### 3.5.2.1 Medically-determined complications

NICU admission rates and respiratory support requirements showed no significant association with MHL after controlling for sociodemographic confounders (31). These decisions are based primarily on objective medical criteria assessed by healthcare providers rather than maternal factors directly influenced by health literacy.

##### 3.5.2.2 Behavior-dependent complications

In stark contrast, conditions requiring maternal recognition, comprehension, and appropriate action showed notably stronger associations with MHL. Maternal health literacy was identified as the sole significant predictor of neonatal jaundice readmissions, with higher MHL associated with reduced readmission risk among Myanmar mothers residing in Thailand (aOR 0.96, 95% CI 0.94–0.98) (39). This association likely reflects enhanced maternal ability to: recognize jaundice progression, comprehend discharge instructions regarding infant monitoring, assess symptom severity, and make appropriate decisions about seeking timely care. Most compellingly, a randomized controlled trial demonstrated that a teach-back intervention specifically targeting MHL significantly reduced diaper dermatitis compared to standard care (37). This experimental evidence provides preliminary support for a relationship between MHL-targeted interventions and infant outcomes requiring maternal caregiving competencies—suggesting that health literacy enhancement may translate to improved infant health outcomes, though this inference requires confirmation from additional RCTs.

This systematic pattern across neonatal complications suggests MHL primarily influences infant outcomes through behavioral pathways requiring maternal agency—including information processing, decision-making quality, symptom recognition, healthcare navigation, and caregiving skill execution—rather than through direct effects on medical/biological factors determined independently of maternal behavior.

This pattern, if confirmed through prospective designs, has implications for intervention design. MHL may demonstrate selective influence on outcomes where maternal capabilities directly matter. Health literacy enhancement strategies may therefore achieve maximum effectiveness when strategically targeted toward outcomes most dependent on maternal behavioral responses.

#### 3.5.3 Domain 3: postnatal growth and development—evidence of extended influence

Although examined in only two studies, postnatal outcomes revealed some of the largest effect magnitudes observed in this review, suggesting that MHL influences may extend well beyond the immediate perinatal period.

##### 3.5.3.1 Nutritional status

A cross-sectional study in both rural and urban resource-constrained settings in India examined children aged 12–23 months. Children of mothers with adequate health literacy had approximately half the likelihood of severe stunting or severe underweight compared to children of mothers with limited health literacy, even after extensive adjustment for household socioeconomic status and other maternal factors (32). These associations likely reflect MHL’s ongoing influence on multiple pathways: feeding practices, dietary diversity, malnutrition recognition, and healthcare-seeking for growth concerns.

##### 3.5.3.2 Developmental outcomes

A US study of unauthorized Mexican immigrant mothers found those with low MHL had more than four times the odds of having children at risk of developmental delay compared to mothers with adequate health literacy (35). This substantial effect magnitude in a vulnerable population suggests MHL may have profound long-term implications for child developmental trajectories, potentially through influences on developmental stimulation, delay recognition, and healthcare engagement for developmental services.

While limited in number, these findings suggest MHL influences extend temporally beyond pregnancy/delivery, continuing to shape child health through ongoing effects on caregiving behaviors, feeding practices, developmental stimulation, and healthcare utilization throughout early childhood. The particularly large effect sizes in vulnerable populations suggest health literacy interventions may be especially impactful for reducing health disparities.

### 3.6 Cross-domain synthesis: MHL as a systemic determinant

Examining patterns across all three outcome domains reveals critical insights about the nature and scope of MHL’s influence on infant and early childhood health:

#### 3.6.1 Breadth of influence

MHL demonstrated associations spanning from immediate birth parameters through acute neonatal complications to longer-term developmental outcomes. This comprehensive scope across the early life course—rather than representing problematic heterogeneity—is consistent with MHL operating as a foundational competency influencing infant health across multiple domains, potentially through shared mechanisms—specifically maternal information processing, decision-making, and caregiving behaviors—that require prospective confirmation.

#### 3.6.2 The behavioral pathway pattern

The most consistent associations emerged for outcomes where maternal information processing, decision-making, and active caregiving directly influence health status:

- *Birth weight*: reflecting cumulative prenatal health behaviors (nutrition, supplementation, substance avoidance).
- *Jaundice readmission*: requiring symptom recognition and timely care-seeking decisions.
- *Diaper dermatitis*: requiring comprehension and execution of proper hygiene practices.
- *Nutritional status*: reflecting ongoing feeding practices and dietary management.
- *Developmental outcomes*: reflecting developmental stimulation provision and service utilization.

Conversely, outcomes determined primarily by medical factors independent of maternal agency showed weaker/null associations, supporting the behavioral pathway hypothesis.

#### 3.6.3 Effect magnitude variation

Where quantitatively reported, effect sizes demonstrated considerable variation, ranging from approximately 30–35% increased risk for birth-related outcomes to over 400% increased risk for developmental delay in vulnerable populations. This variation likely reflects several factors: outcome-specific mechanisms, population vulnerability, and contextual factors. However, where multiple studies are available, effect magnitudes indicate potentially clinically significant associations; single-study findings should be interpreted with caution pending replication.

#### 3.6.4 Population vulnerability

The largest effect magnitudes appeared in socioeconomically disadvantaged and vulnerable populations—unauthorized immigrant mothers facing language barriers and legal status concerns, families in resource-constrained settings with limited healthcare access. This pattern suggests that MHL effects may be most pronounced when families face multiple, compounding challenges, supporting MHL’s potential as an equity-promoting intervention target. When health literacy limitations compound other social disadvantages, the resulting impact on infant health outcomes may be amplified.

## 4 Discussion

### 4.1 Principal findings: MHL as a systemic public health determinant

This systematic review provides the first comprehensive evidence synthesis examining MHL’s associations with the full spectrum of infant and early childhood health outcomes across the first 3 years of life. Drawing from eight studies involving 13,407 participants across seven countries and diverse economic contexts, our findings suggest that MHL may function as a systemic, cross-cutting public health determinant with meaningful associations across multiple outcome domains.

The deliberate inclusion of diverse outcomes—from fetal growth indicators to developmental milestones—is a critical strength that challenges the fragmented perspective of traditional reviews, which often treat MHL as a narrow predictor of isolated behaviors. The most robust evidence emerged for birth weight outcomes, with three studies demonstrating consistent associations between higher MHL and favorable birth weight across diverse populations. Mothers with adequate health literacy showed approximately two-fold increased likelihood of delivering normal-weight infants, with associations persisting after adjustment for traditional socioeconomic confounders including maternal education, income, age, and race/ethnicity. This suggests MHL may operate through partially independent pathways beyond conventional socioeconomic determinants.

### 4.2 The behavioral pathway hypothesis: a hypothesis framework for future investigation

The most conceptually significant contribution is the proposal of a behavioral pathway hypothesis, grounded in a cross-domain pattern of associations that, while currently observational, provides a coherent and testable framework for future investigation. This pattern suggests that MHL’s influence is selective: outcomes that primarily require maternal agency— including nutrition-related behaviors, symptom recognition, and caregiving execution (44–46)—showed significantly stronger and more consistent associations than outcomes determined predominantly by medical or biological factors.

This hypothesis finds preliminary, cross-domain consistency across all three outcome domains examined. For birth outcomes, the consistent associations with birth weight (reflecting cumulative prenatal health behaviors including nutrition, supplementation, and substance avoidance) contrast sharply with null findings for preterm birth and mixed findings for Apgar scores—outcomes more heavily influenced by acute medical circumstances. For neonatal complications, the stark contrast between behavior-dependent and medically-determined outcomes provides compelling evidence: while NICU admission and respiratory support requirements (determined by objective clinical criteria) showed no significant MHL associations, neonatal jaundice readmission (requiring maternal symptom recognition and care-seeking) and diaper dermatitis (requiring caregiving skill execution) showed notably stronger associations, consistent with the behavioral pathway hypothesis. Most compellingly, the sole experimental evidence—a single RCT examining one specific outcome (diaper dermatitis)—preliminarily supports a relationship between MHL-targeted intervention and behavior-dependent infant outcomes; however, the scope of this causal inference is strictly limited to this outcome and setting, and cannot be extrapolated to the broader behavioral pathway hypothesis without further RCT evidence across multiple outcome domains. For postnatal growth and development, although examined in only two studies, the substantial effect magnitudes observed—approximately 50% reduced odds of severe stunting/underweight in India and over four-fold increased developmental delay risk in vulnerable US immigrant populations—suggest MHL influences extend through ongoing caregiving behaviors, feeding practices, and healthcare utilization patterns throughout early childhood.

### 4.3 Public health significance and intervention implications

From a public health investment perspective, the systemic influence pattern of MHL is crucial. The breadth of associations across birth outcomes, neonatal complications, and postnatal development suggests that interventions targeting MHL can yield cascading benefits across multiple health indicators rather than isolated improvements, positioning MHL enhancement as a potentially high-value intervention strategy relative to narrowly-focused single-outcome approaches, though cost-effectiveness claims require confirmation from adequately powered experimental studies.

The concentration of limited MHL among socioeconomically disadvantaged populations (15–45% prevalence across included studies, consistent with global estimates of 30–50% inadequate health literacy), combined with evidence of particularly large effect sizes in vulnerable groups, positions MHL as a promising equity-promoting intervention target. The four-fold increased developmental delay risk observed specifically among unauthorized Mexican immigrant mothers with low health literacy, and the substantial nutritional status associations in resource-constrained Indian settings, suggest that MHL interventions may be especially impactful for reducing health disparities when targeted toward populations facing multiple compounding challenges. When health literacy limitations compound other social disadvantages—language barriers, legal status concerns, limited healthcare access—the resulting impact on infant health outcomes appears amplified.

Translating evidence into action requires a comprehensive multi-level approach. Healthcare systems should consider integrating routine MHL screening (ideally in early pregnancy) coupled with systematic intervention protocols. Evidence-based strategies include teach-back methods, plain language communication eliminating medical jargon, visual aids accommodating diverse learning preferences, and structured care navigation supporting healthcare system engagement. System-level modifications should include redesigning patient materials to appropriate literacy levels, implementing comprehensive multilingual support (particularly important given that several included studies involved immigrant and non-native speaker populations), and training support staff in health literacy-sensitive approaches.

Policymakers must recognize MHL’s role as a modifiable determinant and direct research funding toward methodological advances. Critical priorities include developing standardized, pregnancy-specific MHL assessment tools, conducting adequately powered randomized controlled trials, and implementing prospective longitudinal studies to establish temporal precedence and causal relationships. Integration of health literacy assessment and intervention into maternal care quality standards and clinical practice guidelines would create accountability for addressing this determinant systematically.

### 4.4 Strengths and limitations

This systematic review offers several strengths: first systematic examination of MHL’s associations with actual infant health status (not just maternal behaviors) across the comprehensive early life course (birth to age three); geographic diversity across seven countries spanning diverse economic contexts; large cumulative sample (*n* = 13,407); rigorous PRISMA-compliant methodology with standardized JBI quality assessment and formal GRADE evidence certainty evaluation; and proposal of the behavioral pathway hypothesis, which frames MHL as a potentially systemic determinant warranting further investigation.

However, significant limitations warrant cautious interpretation. Measurement heterogeneity—eight different instruments including only two perinatal-specific tools, of which only one has been formally validated—limits direct comparability and prevents meta-analysis. Cross-sectional design predominance restricts causal inference and cannot establish temporal precedence or rule out reverse causation. Scarcity of experimental evidence (only one RCT) precludes causal conclusions and limits recommendations about effectiveness or cost-effectiveness to the level of hypothesis-generating propositions. Limited outcome-specific replication, particularly for postnatal growth and development, constrains conclusion robustness. Geographic concentration in middle- and high-income countries, combined with the restriction to English-language publications, introduces compounded selection bias: research from low-income countries and non-English-speaking settings—where limited MHL and its consequences may be most pronounced—is systematically underrepresented. This limitation is particularly salient given the review’s equity focus, and future syntheses should explicitly prioritize multilingual searches and targeted outreach to low-income country research networks as a strategic priority. The review protocol was not prospectively registered in PROSPERO, which may affect methodological transparency; however, strict adherence to pre-specified inclusion criteria and the PRISMA reporting framework partially mitigates this limitation. The Low to Very Low certainty of evidence, as determined by our formal GRADE assessment, underscores that the associations identified remain hypothesis-generating rather than confirmatory, reflecting both the predominance of cross-sectional designs and the small number of studies per outcome domain. These limitations collectively underscore the need for prospective longitudinal studies and adequately powered RCTs to better establish the nature and magnitude of MHL’s influence on early childhood health.

## 5 Conclusion

This systematic review provides preliminary evidence suggesting that maternal health literacy may represent a significant, systemic public health determinant of infant and early childhood health outcomes. Rather than functioning as a narrow predictor of isolated behaviors, MHL exhibits meaningful associations across diverse outcome domains—from birth weight through neonatal complications requiring maternal behavioral responses to longer-term developmental trajectories—suggesting its potential operation as a foundational caregiving competency with broad influence across the early life course.

The central conceptual contribution is the Behavioral Pathway Hypothesis: MHL’s effects appear most pronounced for outcomes requiring maternal agency (information processing, symptom recognition, care-seeking, caregiving execution) rather than medically-determined conditions. This pattern, observed consistently across domains and requiring prospective experimental confirmation, suggests that future interventions may benefit from strategically targeting maternal behavioral competencies linked to behavior-dependent outcomes, if this hypothesis is validated through longitudinal and experimental designs.

This convergence of evidence, coupled with the concentration of limited MHL among vulnerable populations, positions MHL enhancement as a promising, modifiable intervention target and a priority for future public health investigation. The breadth of associations suggests interventions can yield cascading benefits across multiple indicators through common mechanisms, though whether this translates to favorable cost-effectiveness requires empirical confirmation. While methodological limitations, particularly the scarcity of experimental designs, restrict definitive causal claims, the potential for MHL interventions to generate broad, compounding improvements for the most vulnerable mothers and infants warrants prioritized, carefully evaluated public health action alongside dedicated research investment.

## Data Availability

The original contributions presented in the study are included in the article/Supplementary material, further inquiries can be directed to the corresponding author.
