# Supplementary material for: Maternal health literacy as a potential determinant of infant and early childhood health: a systematic review
Source: Front Public Health. 2026 Apr 15;14:1743880. doi: 10.3389/fpubh.2026.1743880 (PMC13125130; doi:10.3389/fpubh.2026.1743880)
Supplement: Supplementary file 1 [file Table_1.DOCX]

**Search Strategy**

PUBMED

① (Health Literacy[MeSH Terms]) OR (health literac*[Title/Abstract])

②"pregnancy"[Title/Abstract] OR "pregnant"[Title/Abstract] OR "postpartum"[Title/Abstract] OR "maternal"[Title/Abstract] OR "breastfeeding"[Title/Abstract] OR "prenatal"[Title/Abstract]

③"measurability"[All Fields] OR "measurable"[All Fields] OR "measurably"[All Fields] OR "measure s"[All Fields] OR "measureable"[All Fields] OR "measured"[All Fields] OR "measurement"[All Fields] OR "measurement s"[All Fields] OR "measurements"[All Fields] OR "measurer"[All Fields] OR "measurers"[All Fields] OR "measuring"[All Fields] OR "measurings"[All Fields] OR "measurment"[All Fields] OR "measurments"[All Fields] OR "weights and measures"[MeSH Terms] OR ("weights"[All Fields] AND "measures"[All Fields]) OR "weights and measures"[All Fields] OR "measure"[All Fields] OR "measures"[All Fields] OR ("instrument"[All Fields] OR "instrument s"[All Fields] OR "instrumentation"[MeSH Subheading] OR "instrumentation"[All Fields] OR "instruments"[All Fields] OR "instrumented"[All Fields] OR "instrumenting"[All Fields]) OR ("screener"[All Fields] OR "screeners"[All Fields]) OR ("scale s"[All Fields] OR "scaled"[All Fields] OR "scaling"[All Fields] OR "scalings"[All Fields] OR "weights and measures"[MeSH Terms] OR ("weights"[All Fields] AND "measures"[All Fields]) OR "weights and measures"[All Fields] OR "scale"[All Fields] OR "scales"[All Fields]) OR ("epidemiology"[MeSH Subheading] OR "epidemiology"[All Fields] OR "surveillance"[All Fields] OR "epidemiology"[MeSH Terms] OR "surveilance"[All Fields] OR "surveillances"[All Fields] OR "surveilled"[All Fields] OR "surveillence"[All Fields]) OR "estimat*"[All Fields] OR ("research design"[MeSH Terms] OR ("research"[All Fields] AND "design"[All Fields]) OR "research design"[All Fields] OR "test"[All Fields]) OR ("questionnair"[All Fields] OR "questionnaire s"[All Fields] OR "surveys and questionnaires"[MeSH Terms] OR ("surveys"[All Fields] AND "questionnaires"[All Fields]) OR "surveys and questionnaires"[All Fields] OR "questionnaire"[All Fields] OR "questionnaires"[All Fields]) OR "TOFHLA"[All Fields] OR "Test of Functional Health Literacy in Adults"[All Fields] OR "Single-Item Literacy Screener"[All Fields] OR ("squamous intraepithelial lesions"[MeSH Terms] OR ("squamous"[All Fields] AND "intraepithelial"[All Fields] AND "lesions"[All Fields]) OR "squamous intraepithelial lesions"[All Fields] OR "sils"[All Fields]) OR (("aspect"[All Fields] OR "aspects"[All Fields]) AND ("health literacy"[MeSH Terms] OR ("health"[All Fields] AND "literacy"[All Fields]) OR "health literacy"[All Fields]) AND ("scale s"[All Fields] OR "scaled"[All Fields] OR "scaling"[All Fields] OR "scalings"[All Fields] OR "weights and measures"[MeSH Terms] OR ("weights"[All Fields] AND "measures"[All Fields]) OR "weights and measures"[All Fields] OR "scale"[All Fields] OR "scales"[All Fields])) OR "AAHLS"[All Fields] OR (("maternal health"[MeSH Terms] OR ("maternal"[All Fields] AND "health"[All Fields]) OR "maternal health"[All Fields]) AND ("literacies"[All Fields] OR "literacy"[MeSH Terms] OR "literacy"[All Fields] OR "literacy s"[All Fields]) AND ("inventoried"[All Fields] OR "inventory s"[All Fields] OR "inventorying"[All Fields] OR "personality inventory"[MeSH Terms] OR ("personality"[All Fields] AND "inventory"[All Fields]) OR "personality inventory"[All Fields] OR "inventories"[All Fields] OR "equipment and supplies"[MeSH Terms] OR ("equipment"[All Fields] AND "supplies"[All Fields]) OR "equipment and supplies"[All Fields] OR "inventory"[All Fields]) AND ("pregnancy"[MeSH Terms] OR "pregnancy"[All Fields] OR "pregnancies"[All Fields] OR "pregnancy s"[All Fields])) OR "MHELIP"[All Fields] OR "Health Literacy Questionnaire"[All Fields] OR ("HLQ"[All Fields] AND "Or"[All Fields] AND ("realm"[All Fields] OR "realms"[All Fields])) OR "SALHSA"[All Fields] OR "Newest Vital Sign"[All Fields] OR "NVS"[All Fields] OR "Life Skills Progression Instrument"[All Fields] OR "LSP"[All Fields] OR (("european people"[MeSH Terms] OR ("european"[All Fields] AND "people"[All Fields]) OR "european people"[All Fields] OR "european"[All Fields] OR "europeans"[All Fields]) AND ("health literacy"[MeSH Terms] OR ("health"[All Fields] AND "literacy"[All Fields]) OR "health literacy"[All Fields]) AND "tool"[All Fields]) OR "HL-EU"[All Fields]

④ ①and ② and ③

Results 672 date March 5, 2025

Embase

①'health literacy'/exp OR 'health literac*'

②pregnancy OR pregnant OR postpartum OR maternal OR breastfeeding OR prenatal

③measurement OR instrument OR screener OR scale OR surveillance OR estimat* OR test OR questionnaire OR tofhla OR 'test of functional health literacy in adults' OR 'single-item literacy screener' OR sils OR 'all aspects of health literacy scale' OR aahls OR 'maternal health literacy inventory in pregnancy' OR mhelip OR 'health literacy questionnaire' OR hlq OR realm OR salhsa OR 'newest vital sign' OR nvs OR 'life skills progression instrument' OR lsp OR 'european health literacy tool' OR 'hl eu'

④ ①and ② and ③

Results 823 date March 5, 2025

Web of Science

① ts=Health Literac*

②ts=pregnancy or ts=pregnant or ts=postpartum or ts=maternal or ts=breastfeeding or ts=prenatal

③ts=measurement or ts=instrument or ts=Screener or ts=scale or ts=surveillance or ts=Estimat* or ts=Test or ts=questionnaire or ts=TOFHLA or ts=(Test of Functional Health Literacy in Adults) or ts=(Single-Item Literacy Screener) or ts=SILS or ts=(All Aspects of Health Literacy Scale) or ts=AAHLS or ts=(Maternal health literacy inventory in pregnancy) or ts=MHELIP or ts=(Health Literacy Questionnaire) or ts=HLQ Or ts=REALM or ts=SALHSA or ts=(Newest Vital Sign) or ts=NVS or ts=(Life Skills Progression Instrument) or ts=LSP or ts=(European Health Literacy Tool) or ts=HL-EU

④ ①and ② and ③

Results 1212 date March 5, 2025
