# Supplementary material for: Maternal health literacy as a potential determinant of infant and early childhood health: a systematic review
Source: Front Public Health. 2026 Apr 15;14:1743880. doi: 10.3389/fpubh.2026.1743880 (PMC13125130; doi:10.3389/fpubh.2026.1743880)
Supplement: Supplementary file 2 [file Table_2.DOCX]

Appendix 2 assessment of the methodological quality by JBI-MAStARI

| Reference | Q1 | Q2 | Q3 | Q4 | Q5 | Q6 | Q7 | Q8 | Total |
| --- | --- | --- | --- | --- | --- | --- | --- | --- | --- |
| Hernandez-M R *et al.*  (2016) | Y | Y | N | Y | N | N | Y | Y | 5 |
| Johri M *et al*.  (2016) | y | y | N | Y | Y | N | Y | Y | 6 |
| Izadirad H *et al.*  (2021) | y | y | Y | Y | N | N | Y | Y | 6 |
| Yee LM *et al.*  (2021) | y | y | N | Y | Y | N | Y | Y | 6 |
| Gaupšienė A *et al.*  (2023) | y | y | N | Y | N | N | Y | Y | 6 |
| Rahimli OS *et al.*  (2024) | y | y | Y | Y | N | N | Y | Y | 6 |
| Eksamut W *et al.*  (2025) | y | y | Y | Y | Y | N | Y | Y | 7 |

N, No; U, Unclear; Y, Yes.

Q1. Were the criteria for inclusion in the sample clearly defined? Q2. Were the study subjects and the setting described in detail? Q3. Was the exposure measured in a valid and reliable way? Q4. Were objective, standard criteria used for measurement of the condition? Q5. Were confounding factors identified? Q6. Were strategies to deal with confounding factors stated? Q7. Were the outcomes measured in a valid and reliable way? Q8. Was appropriate statistical analysis used?

Table assessment of the methodological quality by JBI Critical appraisal checklist for randomized controlled trials

| Reference | Q1 | Q2 | Q3 | Q4 | Q5 | Q6 | Q7 | Q8 | Q9 | Q10 | Q11 | Q12 | Q13 | Total |
| --- | --- | --- | --- | --- | --- | --- | --- | --- | --- | --- | --- | --- | --- | --- |
| Cheng GZ *et al.*  (2023) | Y | U | Y | U | N | U | Y | Y | Y | Y | Y | Y | Y | 9 |

N, No; U, Unclear; Y, Yes.

Q1. Was true randomization used for assignment of participants to treatment groups? Q2. Was allocation to treatment groups concealed? Q3. Were treatment groups similar at the baseline? Q4. Were participants blind to treatment assignment? Q5. Were those delivering treatment blind to treatment assignment? Q6. Were outcomes assessors blind to treatment assignment? Q7. Were treatment groups treated identically other than the intervention of interest? Q8. Was follow up complete and if not, were differences between groups in terms of their follow up

adequately described and analyzed? Q 9. Were participants analyzed in the groups to which they were randomized? Q 10. Were outcomes measured in the same way for treatment groups? Q 11. Were outcomes measured in a reliable way? Q 12. Was appropriate statistical analysis used? Q13. Was the trial design appropriate, and any deviations from the standard RCT design (individual

randomization, parallel groups) accounted for in the conduct and analysis of the trial?
