# Supplementary material for: Maternal health literacy as a potential determinant of infant and early childhood health: a systematic review
Source: Front Public Health. 2026 Apr 15;14:1743880. doi: 10.3389/fpubh.2026.1743880 (PMC13125130; doi:10.3389/fpubh.2026.1743880)
Supplement: Supplementary file 3 [file Table_3.DOCX]

Appendix 3 detailed GRADE assessment

| **Outcome** | **No. of Studies** | **Study Design** | **Risk of Bias** | **Inconsistency** | **Indirectness** | **Imprecision** | **Publication Bias** | **Overall Certainty** |
| --- | --- | --- | --- | --- | --- | --- | --- | --- |
| **Birth weight** | 3 | Observational | Not serious | Not serious | Not serious | Serious | Undetected | ⊕⊕ Low |
| **Apgar scores** | 3 | Observational | Serious | Serious | Not serious | Serious | Undetected | ⊕ Very Low |
| **Diaper dermatitis** | 1 | RCT | Serious | Not serious | Not serious | Serious | Undetected | ⊕⊕ Low |
| **Jaundice readmission** | 1 | Observational | Serious | Not serious | Not serious | Very Serious | Undetected | ⊕ Very Low |
| **Nutritional status** | 1 | Observational | Serious | Not serious | Not serious | Very Serious | Undetected | ⊕ Very Low |
| **Developmental delay** | 1 | Observational | Serious | Not serious | Not serious | Very Serious | Undetected | ⊕ Very Low |
